# Supplementary figures and images for: Suppressor effect of catechol-O-methyltransferase gene in prostate cancer
Source: PLoS One. 2021 Sep 29;16(9):e0253877. doi: 10.1371/journal.pone.0253877 (PMC8480839; doi:10.1371/journal.pone.0253877)

Figure 1G

S-COMT

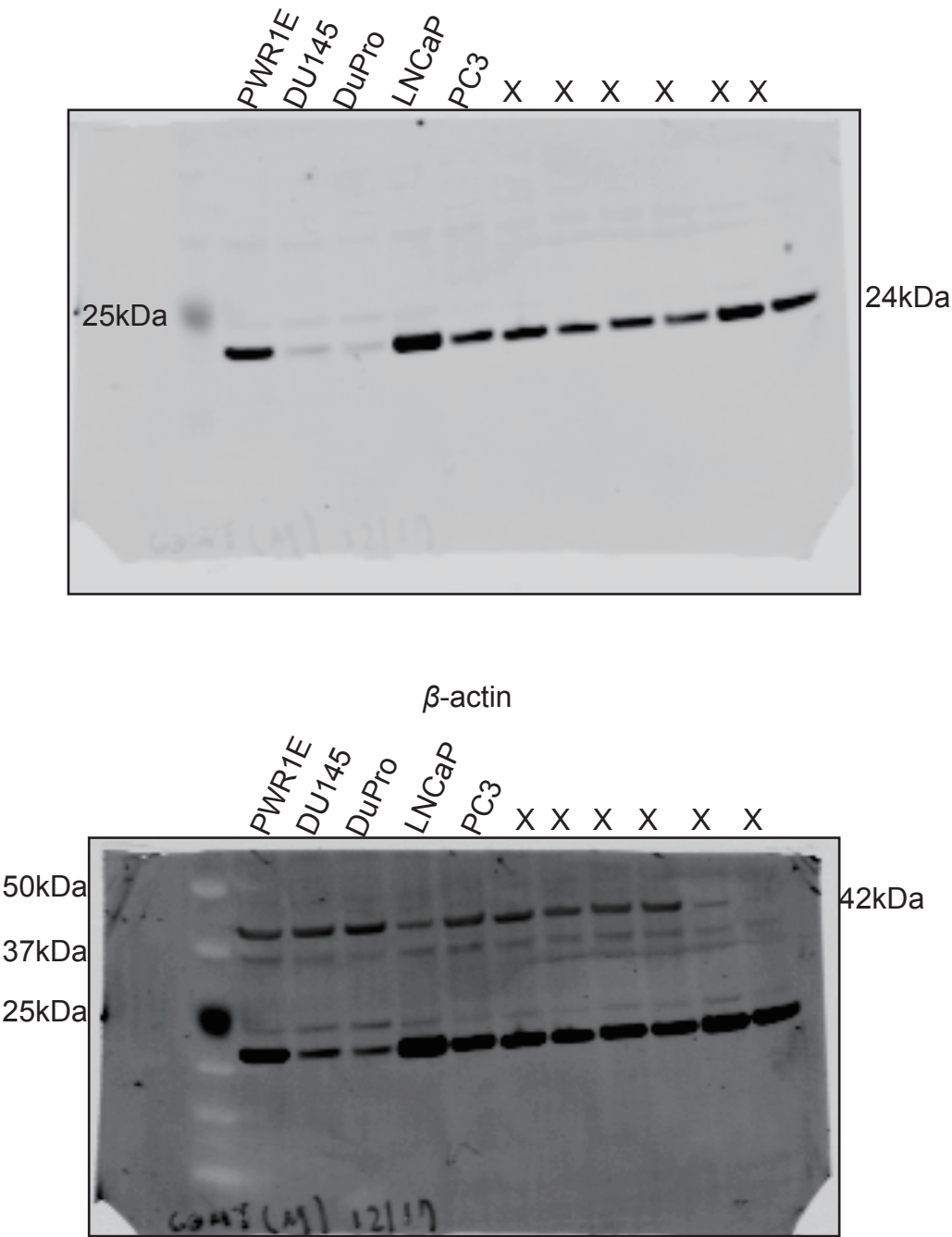

Figure 2A

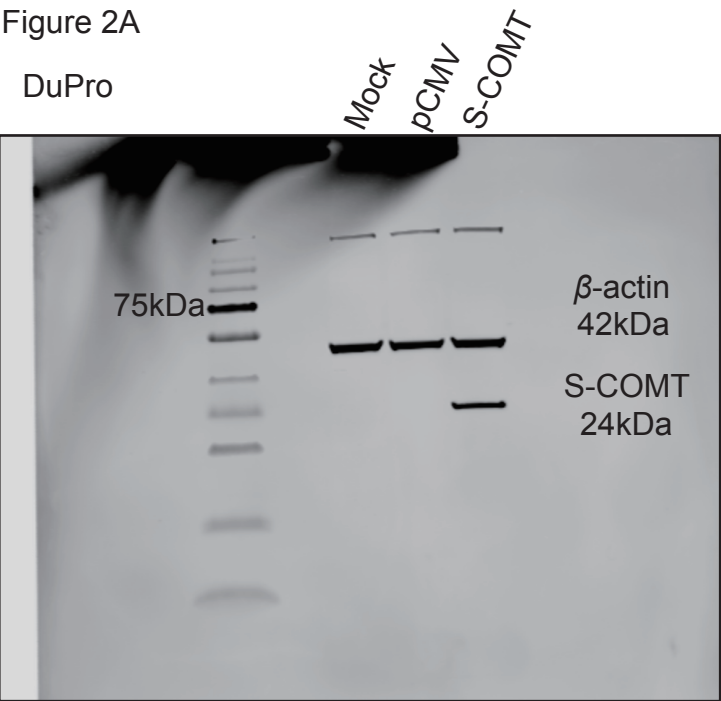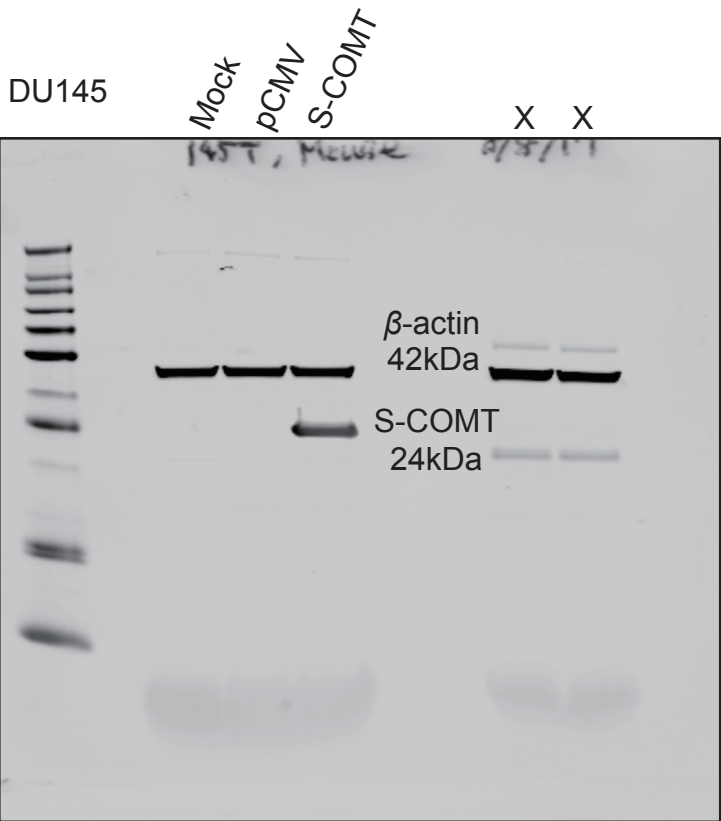

Figure 4

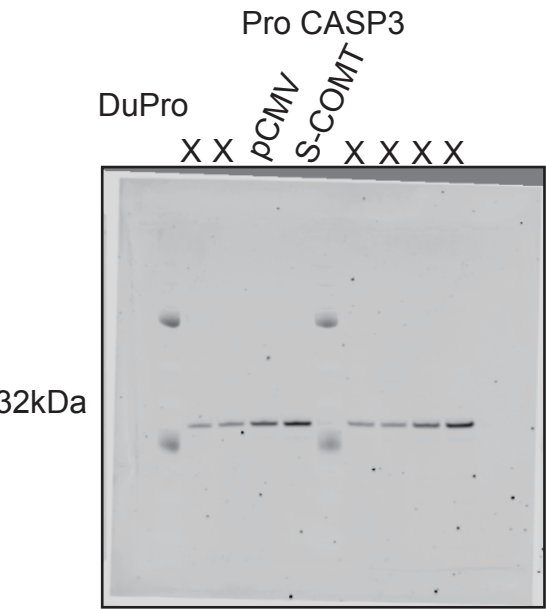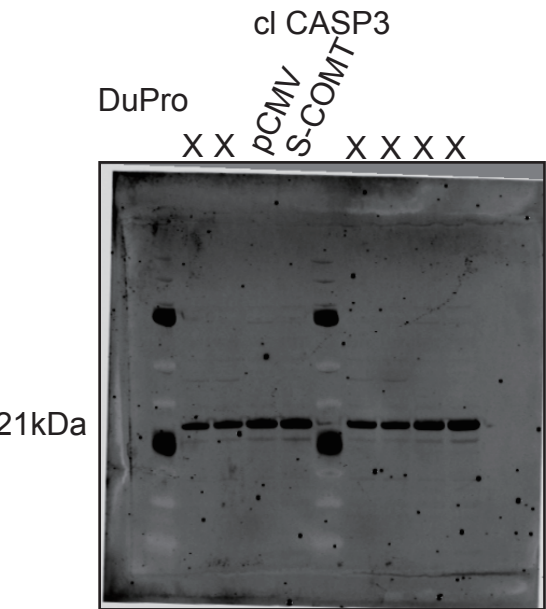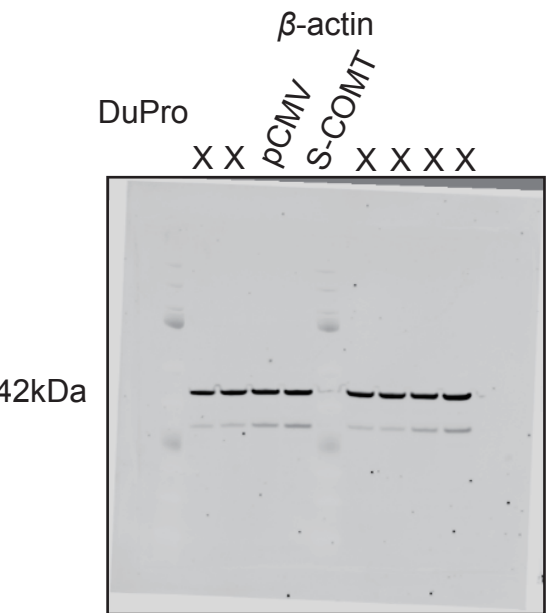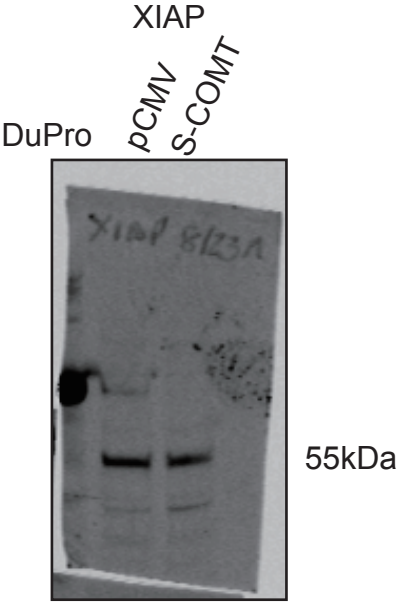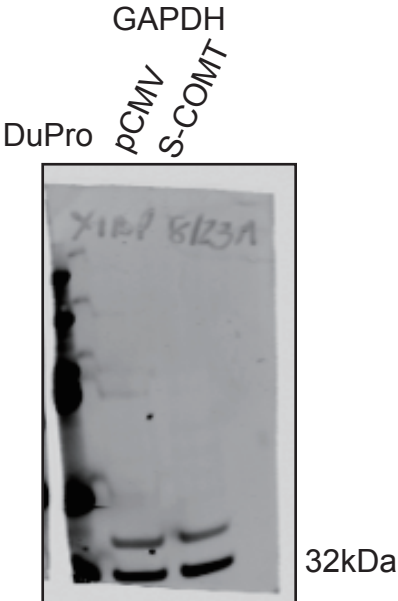

Figure 4 continued

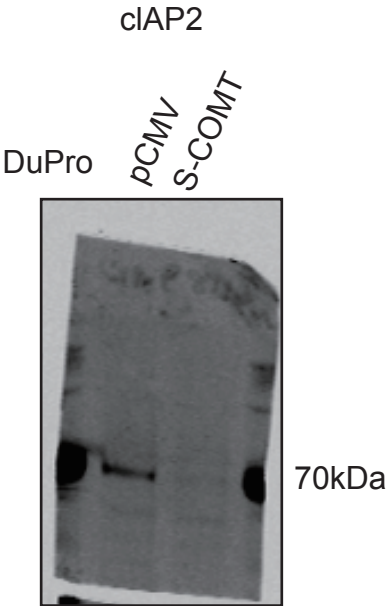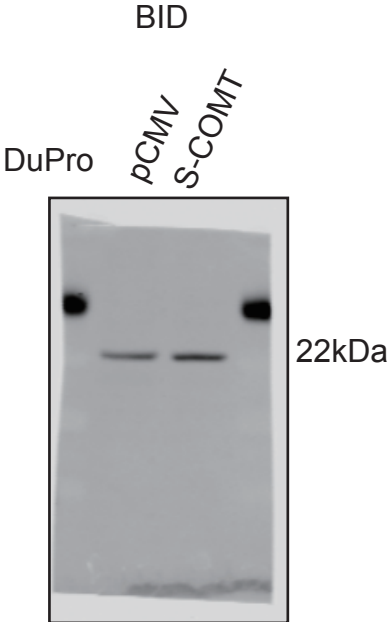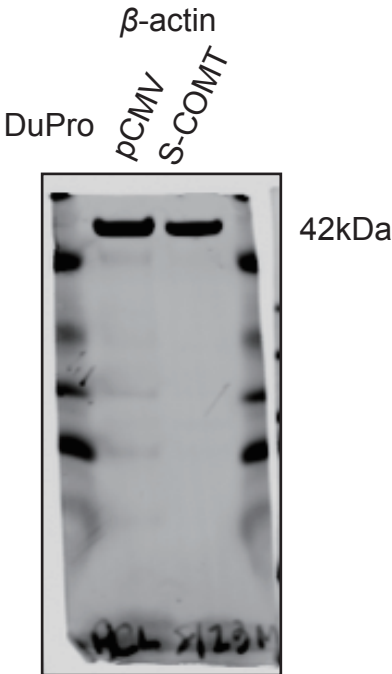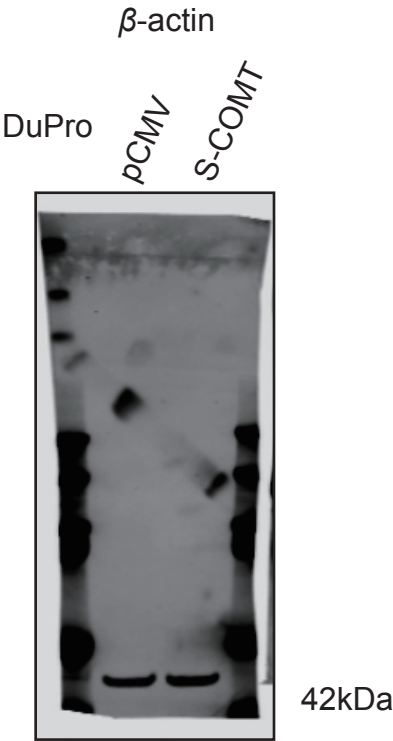

Figure 6C

S-COMT

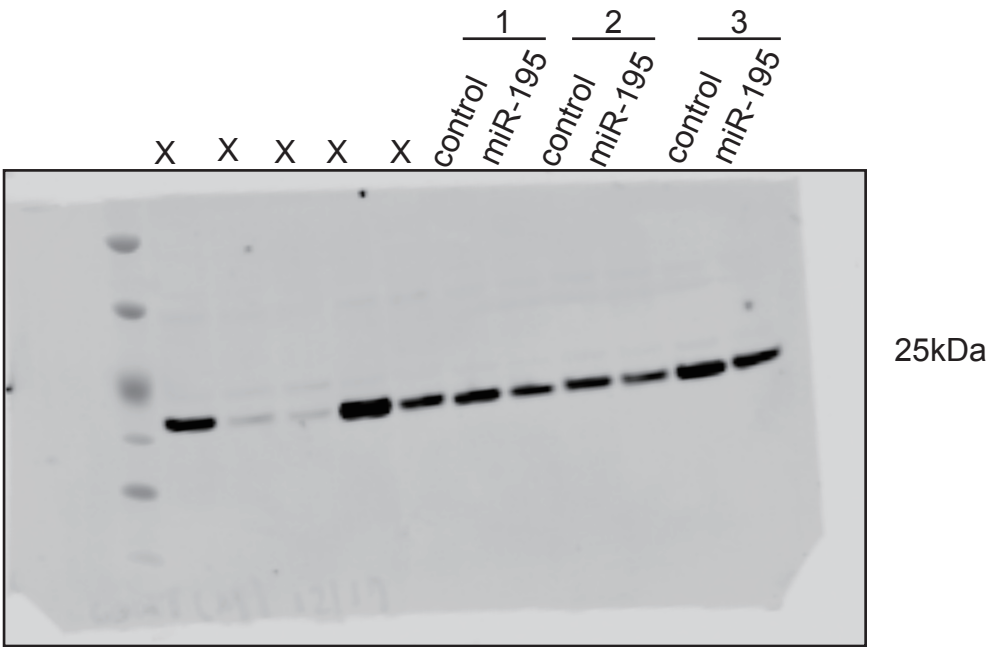

- 1. PWR-1E
- 2. PC3
- 3. LNCaP

$\beta$ -actin

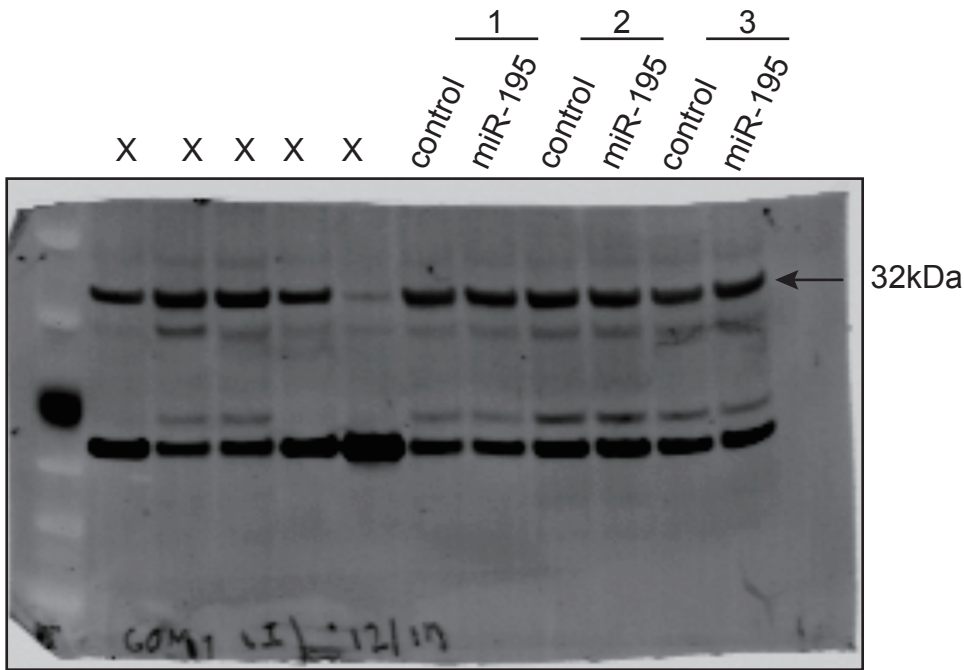

Supplement: S1 Fig — (PDF) [file pone.0253877.s001.pdf]
