# Supplementary material for: Suppressor effect of catechol-O-methyltransferase gene in prostate cancer
Source: PLoS One. 2021 Sep 29;16(9):e0253877. doi: 10.1371/journal.pone.0253877 (PMC8480839; doi:10.1371/journal.pone.0253877)
Supplement: S2 Fig — (PDF) [file pone.0253877.s002.pdf]

S2 Figure

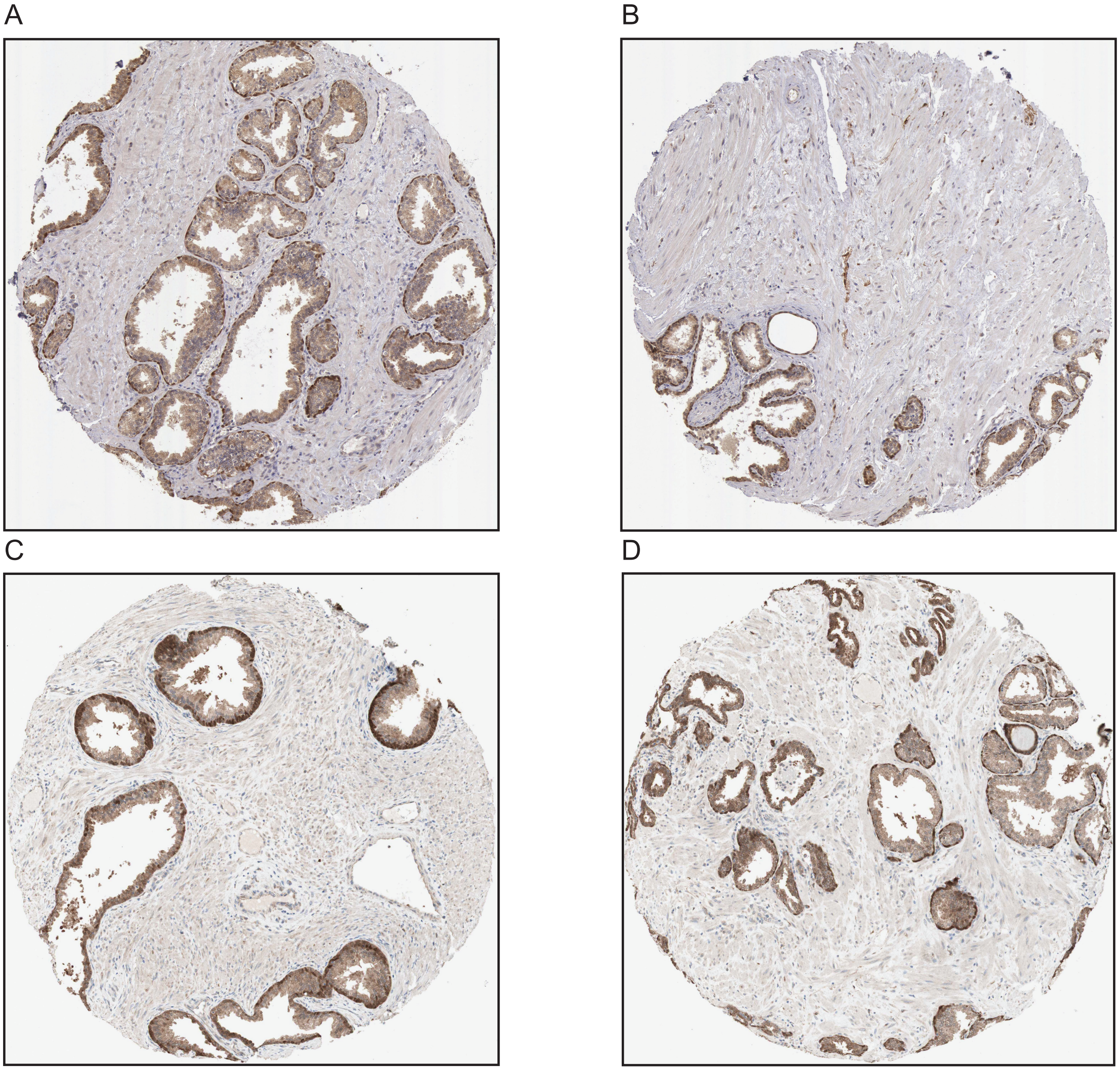

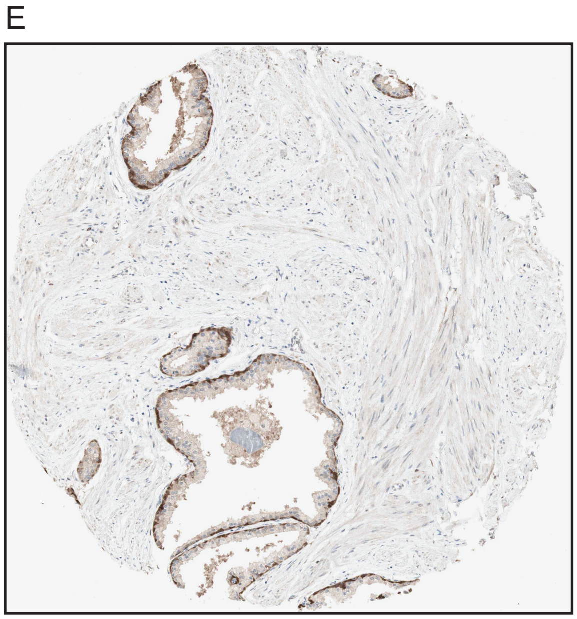

COMT expression was detected in human normal prostate glandular cells. The five individual normal prostate tissue samples were stained using antibody, "HPA001169" (A)&(B) and CAB011233 (C)-(E). The original data is available in the Protein Atlas (https://www.proteinatlas.org/ENSG00000093010-COMT/tissue/prostate).
