# Supplementary material for: Suppressor effect of catechol-O-methyltransferase gene in prostate cancer
Source: PLoS One. 2021 Sep 29;16(9):e0253877. doi: 10.1371/journal.pone.0253877 (PMC8480839; doi:10.1371/journal.pone.0253877)

S3 Figure. The magnified IHC images in Fig. 1D

BPH

#1: 38.6

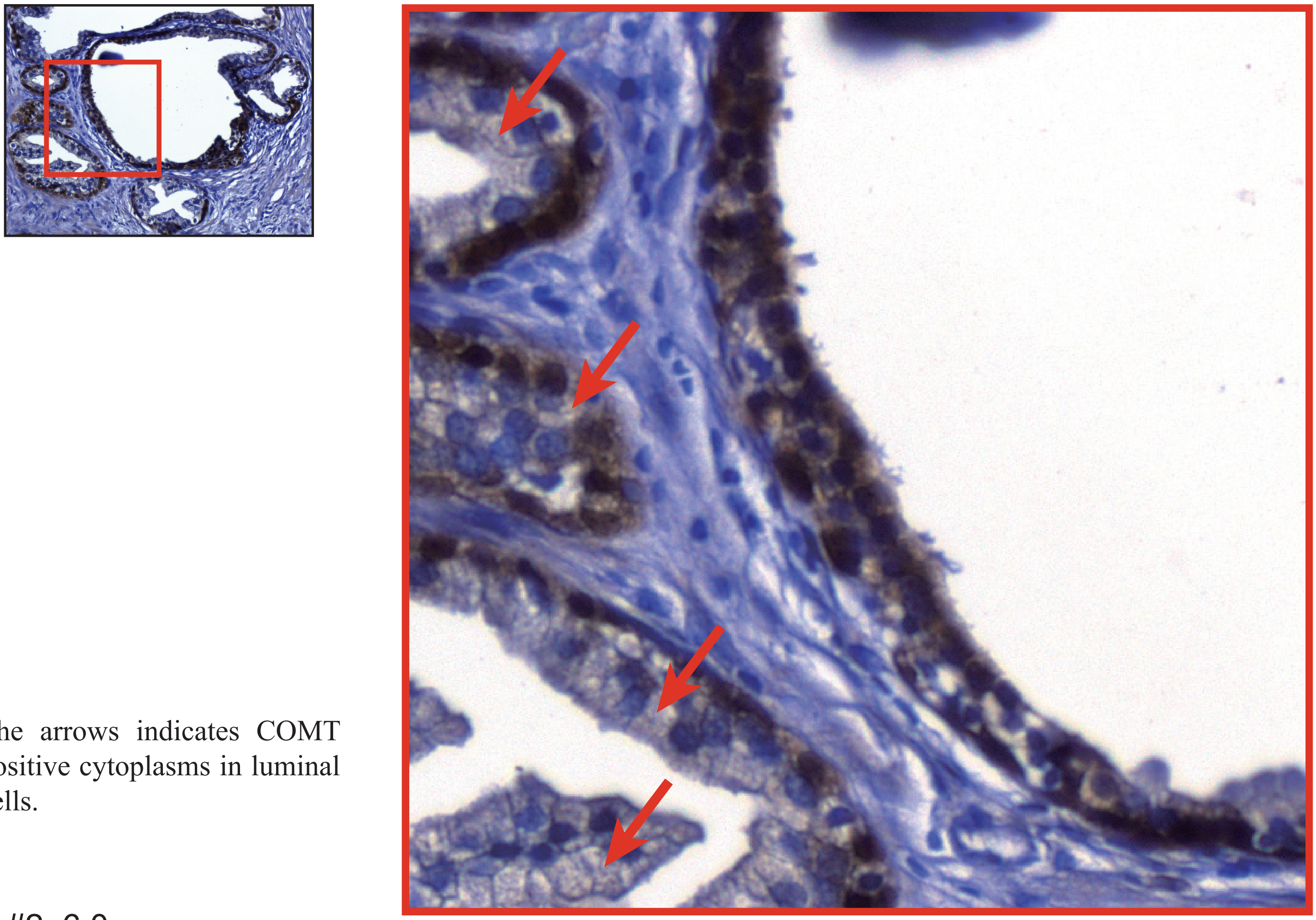

The arrows indicates COMT positive cytoplasms in luminal cells.

#2: 6.0

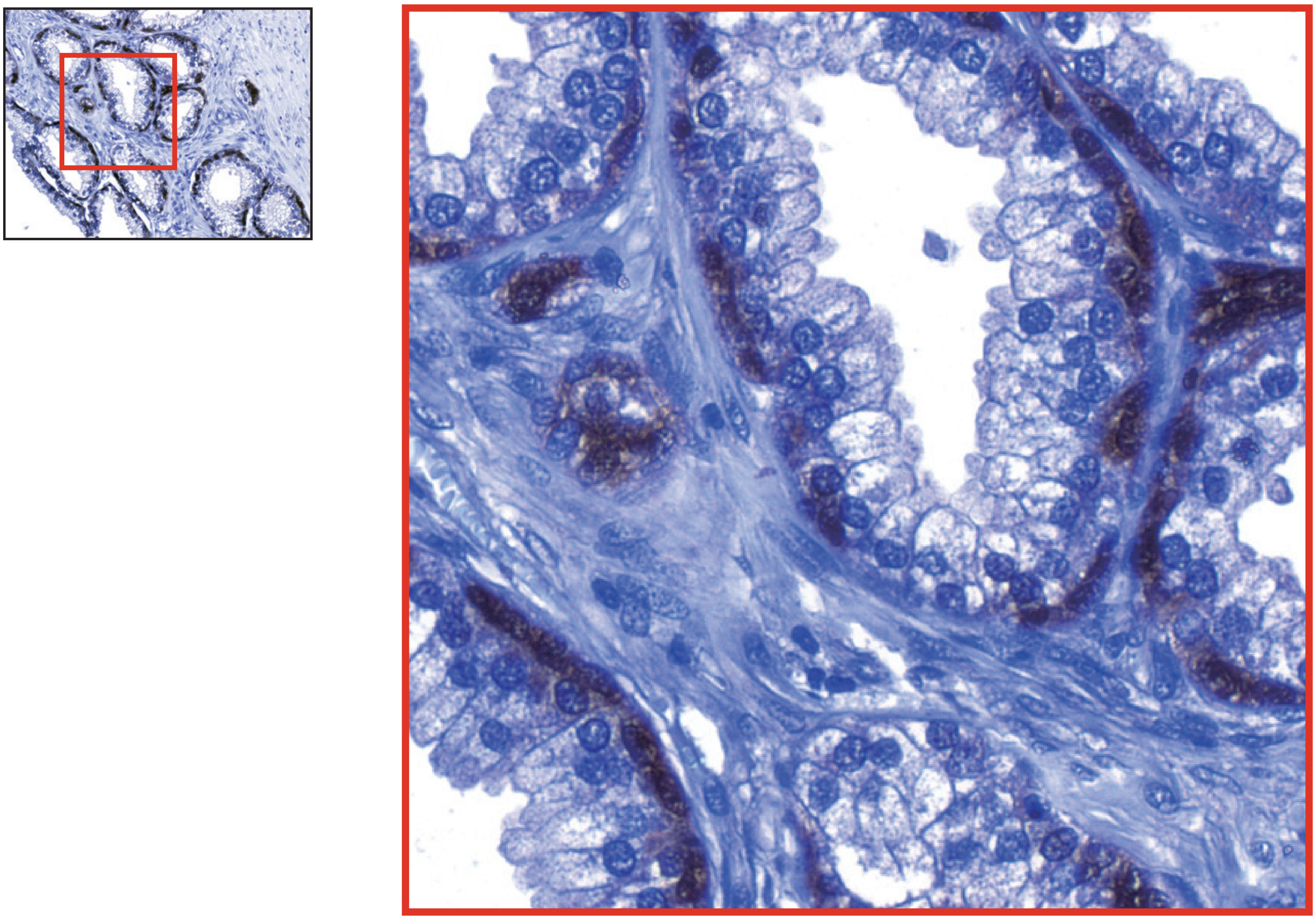

S3 Figure continued. The magnified IHC images in Fig. 1D

PCa

#3: 0.4

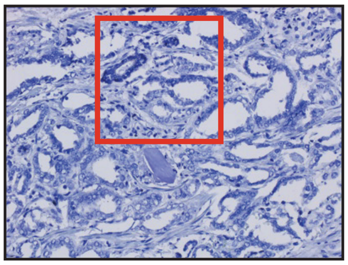

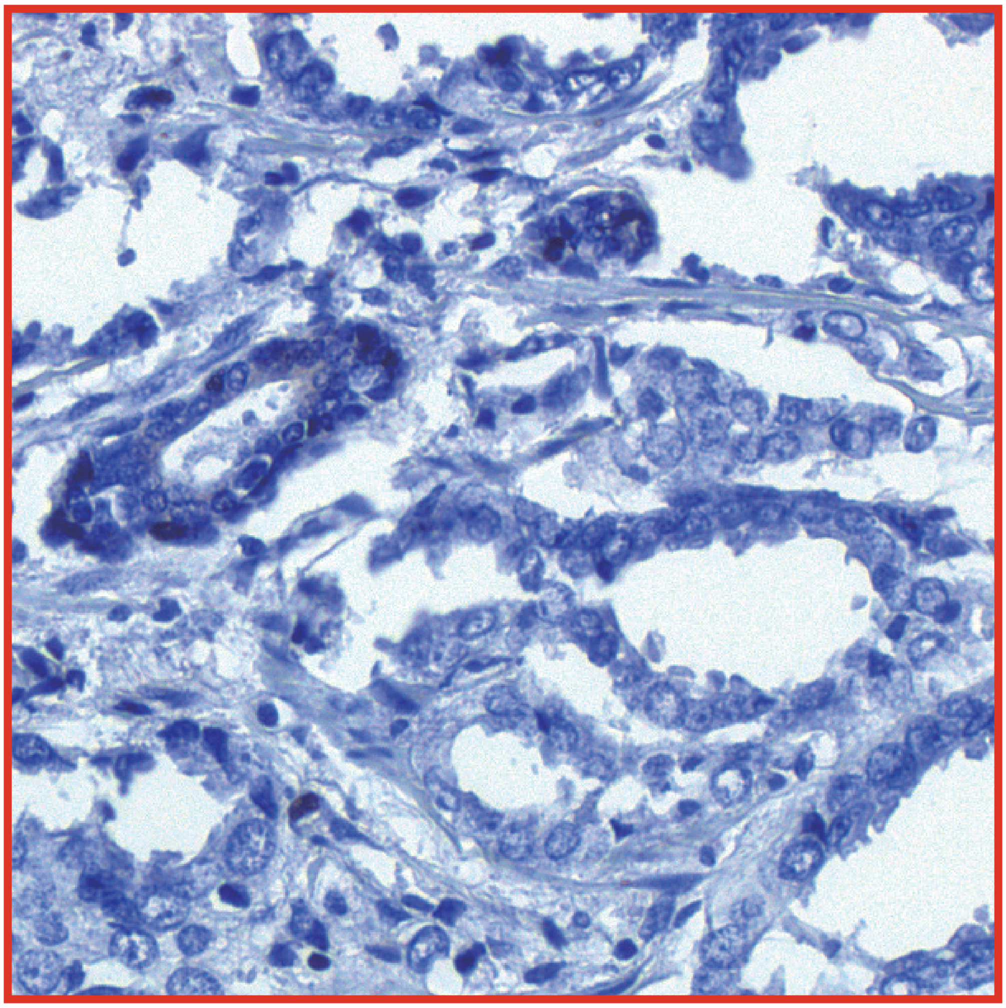

#4: 0.0

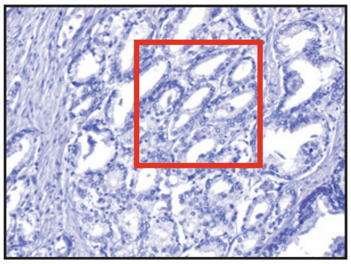

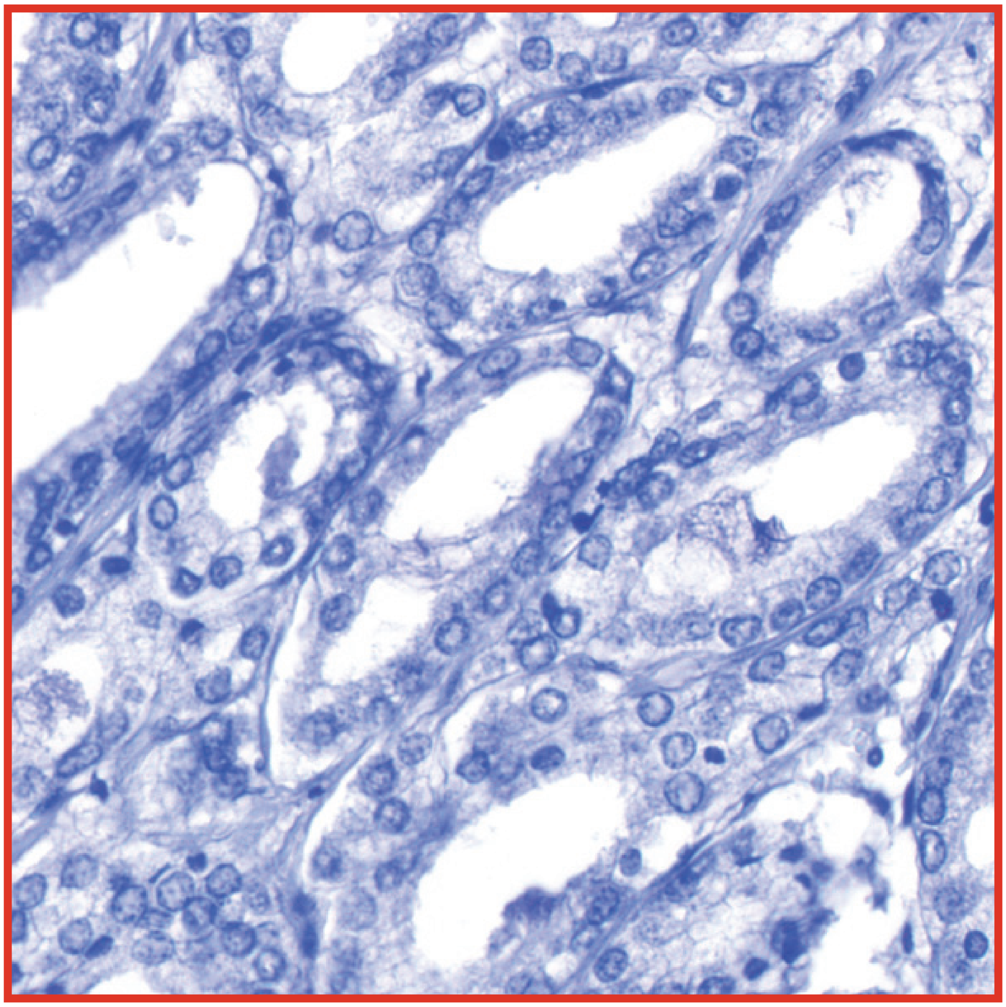

Supplement: S3 Fig — (PDF) [file pone.0253877.s003.pdf]
