# Supplementary material for: Suppressor effect of catechol-O-methyltransferase gene in prostate cancer
Source: PLoS One. 2021 Sep 29;16(9):e0253877. doi: 10.1371/journal.pone.0253877 (PMC8480839; doi:10.1371/journal.pone.0253877)
Supplement: S4 Fig — (PDF) [file pone.0253877.s004.pdf]

S4 Figure. COMT knockdown reduced apoptosis and increased migration in LNCaP cell.

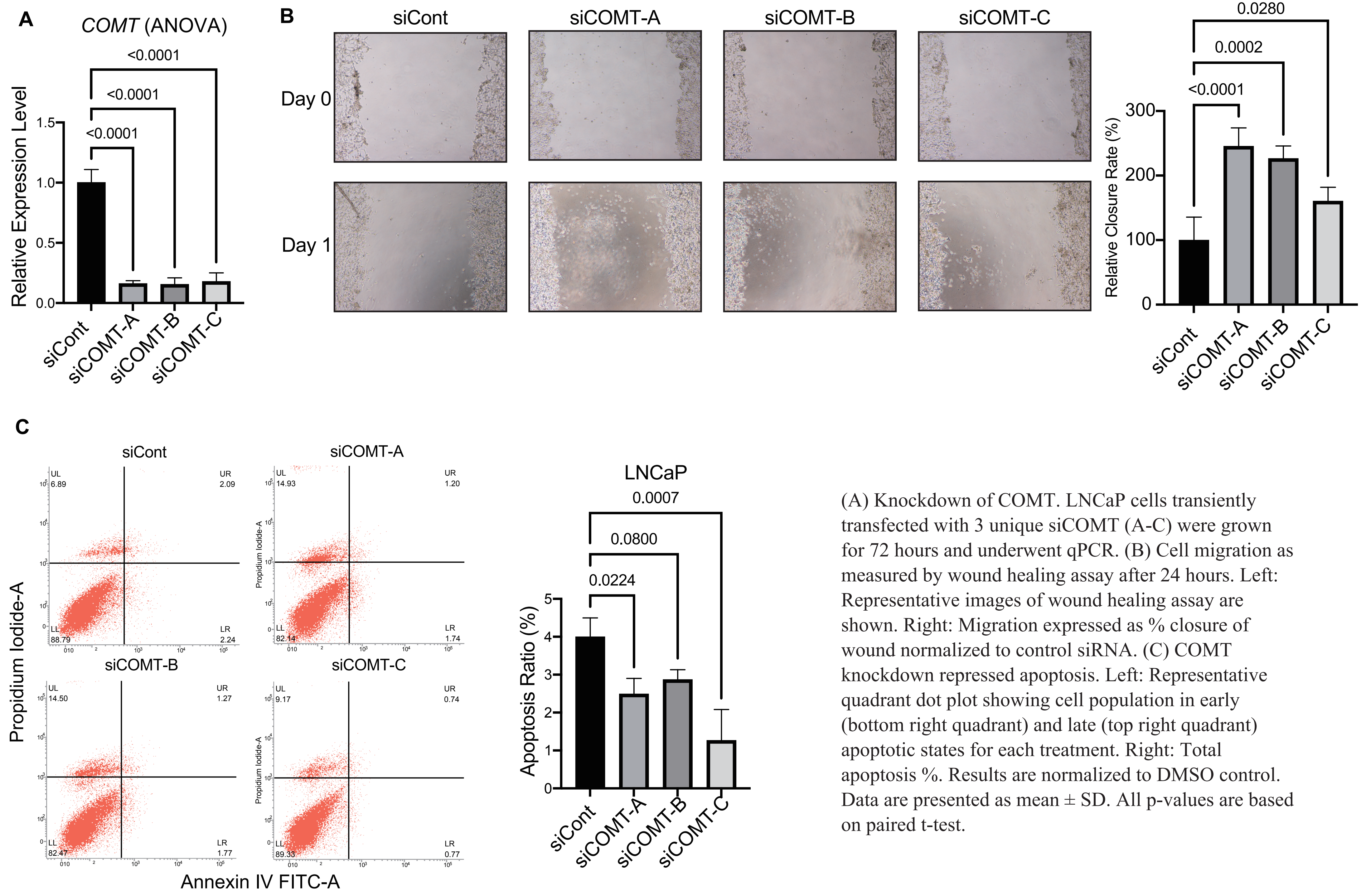

(A) Knockdown of COMT. LNCaP cells transiently transfected with 3 unique siCOMT (A-C) were grown for 72 hours and underwent qPCR. (B) Cell migration as measured by wound healing assay after 24 hours. Left: Representative images of wound healing assay are shown. Right: Migration expressed as % closure of wound normalized to control siRNA. (C) COMT knockdown repressed apoptosis. Left: Representative quadrant dot plot showing cell population in early (bottom right quadrant) and late (top right quadrant) apoptotic states for each treatment. Right: Total apoptosis %. Results are normalized to DMSO control. Data are presented as mean ± SD. All p-values are based on paired t-test.
