# Supplementary material for: Suppressor effect of catechol-O-methyltransferase gene in prostate cancer
Source: PLoS One. 2021 Sep 29;16(9):e0253877. doi: 10.1371/journal.pone.0253877 (PMC8480839; doi:10.1371/journal.pone.0253877)
Supplement: Attachment — Submitted filename: response.docx [file pone.0253877.s005.docx]

**Point by point response to reviewer’s comments**

***Additional Editor* Comments:** The authors should repeat the key experiments in androgen receptor-positive cells and improve quality of images in the manuscript. They should also clarify whether overexpression of COMT has functional implications.

**Response:** We evaluated COMT levels in AR+ LNCaP cells and observed positive COMT expression (Figure 1F,G). Since COMT was expressed, we therefore performed COMT knockdown instead and after evaluating functional effects, we observed results that were consistent with COMT overexpression (Figure S2). We also added better quality images of immunohistochemical results (Figure 1E) and provide an expanded region of staining in Figure S1.

***Reviewer #1***

**Comment 1:** In Fig. 2A, is the COMT overexpression level physiologically relevant?

**Response:** We also performed COMT knockdown in LNCaP cells to evaluate physiological relevance, see S2 Figure.

**Comment 2:** Does COMT knockdown affect proliferation, migration, and/or apoptosis of prostatic cells?

**Response:** We added new S2 Figure that investigated the impact COMT knockdown has on apoptosis and migration of LNCaP cells.

**Comment 3:** In this study, AR-negative cells were used. However, prostate cancer cells in the clinical specimens are in general AR-positive. The authors should also use AR-positive prostate cancer cells.

**Response:** We have measured COMT levels in AR+ PCa cells (LNCaP) and they showed positive COMT expression. For this reason, we could not perform overexpression using AR+ cells. Thus in these LNCaP cells, we now show effects of COMT silencing on functional effects in S2 Figure.

**Comment 4:** In Fig. 6B, the authors showed miR-195 suppression of COMT expression via its 3’-UTR using luciferase assay. It will be more convincing to show that miR-195 knockdown and/or overexpression can indeed modulate the endogenous COMT expression.

**Response:** We performed miR-195 overexpression by using miR-195 mimic in PWR1E, LNCaP and PC3 cells and confirmed that miR-195 mimic could decrease COMT expression, see Figure 6C.

***Reviewer #2***

**Comment 1:** First sentence “…for men during senescence…” - rephrase as “with aging” or “among aging men” or something similar, for clarity.

**Response:** We thank this comment. We edited lines 44-49 since the editorial office also requested some modification on these sentences.

**Comment 2:** Include a reference for DuPro cells in the materials and methods as this is not a commonly used line.

**Response:** A new reference entitled, “Establishment and characterization of a new human prostatic carcinoma cell line (DuPro-1)”, was added as ref. #20 in this version. See line 103.

**Comment 3:** Please include IHC assay positive and negative controls (cells or tissues validated via a different means to contain and not contain COMT).

**Response:** Thank you very much for pointing this out. We did not clearly mention but we used same antibody (Abcam, #ab185954) for western blotting and IHC. Thus, each negative and positive control would be DU145 and PWR1E in Fig 1G. The detailed product information was added in lines 120 and 150 to reconcile the confusion. Also, I would like to point out that picture #1 in Fig 1E was replaced with the sample that shows the highest COMT expression level in BPH samples. This picture #1 is an example of positive COMT staining with a score value of 38.6 whereas picture #4 is an example of negative staining with a score value of 0. We performed IHC because detecting protein is informative whereas measuring longer mRNAs are challenging using FFPE samples.

**Comment 4:** Figure 1 – it would also be of interest to see COMT analyzed in relation to Gleason grade.

**Response:** We added a new boxplot in Fig 1C. We divided into ≤GS3+4 versus ≥GS4+3 which gave a more even n size using TCGA PRAD samples. We found that COMT expression levels are slightly reduced in higher GS compared to lower GS group.

**Comment 5:** It’s unclear why BPH was used as a comparison to prostate cancer. Benign, normal appearing prostate in cases with prostate cancer would have been more informative, and should be included if possible. The IHC images provided in Figure 1D are not at a high enough power (along with low resolution images) to see what the staining pattern is. It looks like possibly some (or all?) of the staining is basal cell staining which obviously would not be applicable to prostate cancer, but it’s impossible to tell with the images presented. Higher power images should be included. Ideally, IHC images of normal prostate (e.g. normal appearing benign regions from radical prostatectomy) should be included.

**Response:** As per reviewer’s comment, we replaced images with higher resolution images and observed COMT staining in luminal cells and basal region. and rephrased the sentence (line 239). Also, magnified images are included in Figure S1. Since the merged PDF file is using lower resolution, please click the link “Click here to access/download;Figure;FigX.tif” at the corner of figure page and the original higher resolution files will appear. Unfortunately, normal region FFPE samples were not available and these are the pictures that we have.

**Comment 6:** If forced expression on COMT triggers apoptosis, this alone could explain the reduced migration and lack of growth in xenograft studies. It’s not really “suppression of tumor growth” so much as triggering of cell death? Please revise the conclusions based on these assays accordingly.

**Response:** Thank you very much for this comment. We modified sentences, see line 430.

**Comment 7:** Line 344/345 – change “significantly oncogenic” to upregulated in cancer compared to normal prostate (or something similar, for clarity).

**Response:** We replace this phrase along with reviewer’s comment. See line 333.

**Comment 7:** Conclusions (also in abstract) – why would COMT be a “therapeutic target” in prostate cancer if it is absent in prostate cancer?

**Response:** Appreciated for pointing this error out. We removed “target” and replace with possible gene for therapeutic development. See line 41 and 432.
