# Supplementary material for: Suppressor effect of catechol-O-methyltransferase gene in prostate cancer
Source: PLoS One. 2021 Sep 29;16(9):e0253877. doi: 10.1371/journal.pone.0253877 (PMC8480839; doi:10.1371/journal.pone.0253877)
Supplement: Attachment — Submitted filename: comment2 - final.docx [file pone.0253877.s006.docx]

**Point by point response to reviewer’s comments**

***Reviewer #2*:**

**COMMENT**: The response to this reviewer's comments are very much appreciated however there is one critical point that has not been adequately addressed. The IHC images shown in Fig. 1E and S1 (which is labeled as S2 in the figure) are much clearer (thank you) but appear to demonstrate that COMT may be limited to prostate atrophy and basal cells and is completely absent in normal luminal prostate epithelium and prostate cancer. The cancer region magnified in Fig S1 does not demonstrate any cancer cell staining. The authors need to provide IHC evidence that COMT is actually expressed in normal appearing luminal epithelium and (presumably to a lesser extent) in prostate cancer cells if that is indeed the case as much of the premise of this manuscript is based on this.

**RESPONSE**: Thank you very much for your comment. As mentioned before, we have only samples donated from patients who went through radical prostatectomy. Thus, we could not obtain the normal tissue samples. Instead, we provided the link and a picture from the database Human Protein Atlas that displays human normal prostate tissues stained with COMT antibodies. COMT expression was detected in the cytoplasm and nucleus of glandular cells (luminal and basal cells). We now add arrows in Figure S1 which point to COMT-positivity in luminal cells, as seen in tissues in Protein Atlas. In the Discussion section, we discuss this matter and also add references that demonstrate basal cell transformation to be involved in prostate carcinogenesis (lines 385-393).
